# Redox-dependent protein S-glutathionylation governs azacitidine sensitivity and resistance in AML

**DOI:** 10.1016/j.redox.2025.103958

**Published:** 2025-12-04

**Authors:** Dušan Nemes, Michaela Myšáková, Lubomír Minařík, Anna Jonášová, Tomáš Stopka, Kristýna Gloc Pimková

**Affiliations:** a BIOCEV, First Faculty of Medicine, Charles University, Vestec, 25250, Czech Republic; b Clinic Haematology, General Faculty Hospital, Prague, 12808, Czech Republic; c Faculty of Science, Charles University, Prague, 128 00, Czech Republic

**Keywords:** Drug resistance, Hypomethylation therapy, Azacitidine, S-glutathionylation, Cysteine oxidation, Redox proteomics, Acute myeloid leukemia, Glyoxalase system, DNA damage

## Abstract

Disruption of redox metabolism is a hallmark of drug-resistant cancer cells, representing a major obstacle to the effective treatment of acute myeloid leukemia (AML). While recent studies have highlighted the importance of redox balance in AML therapy, the specific contribution of protein redox signaling to resistance remains poorly understood. Defining these mechanisms could uncover therapeutic vulnerabilities of resistant AML cells and guide the development of novel combination strategies. Here, we performed comprehensive mass spectrometry–based redox and quantitative proteomic profiling of AML cell lines and patient samples sensitive or resistant to the hypomethylating agent azacitidine (AZA). We demonstrate that AZA disrupts redox homeostasis, which inactivates the glyoxalase system and DNA damage response, and thereby induces cell death. In contrast, AZA resistance is associated with a redox reset characterized by elevated glutathione levels and diminished protein S-glutathionylation. Importantly, AZA failed to induce oxidation of proteins in these pathways in resistant cells and patient-derived AML samples. Pharmacological inhibition of glutathione synthesis restored protein S-glutathionylation and resensitized resistant AML cells to AZA.

## Highlights

• Azacitidine induces ROS generation and protein S-glutathionylation in sensitive AML cells, targeting redox-sensitive cysteine residues.
• Selective oxidation of DNA repair proteins (PARP1) and metabolic enzymes (GLO1) leads to DNA damage and apoptosis as key components of azacitidine's mechanism of action.
• Resistant cells evade AZA toxicity by maintaining a high GSH/GSSG ratio, upregulating glutaredoxin, and reducing global S-glutathionylation.
• Glutathione depletion resensitizes resistant cells to AZA, while GSH supplementation protects sensitive cells, underscoring the central role of GSH metabolism.
• The redox signature of primary AZA-relapsed cells aligned with azacitidine-resistant lines, implicating the mitochondrial and glyoxalase system in resistance.

## Graphical abstract

**Figure:**
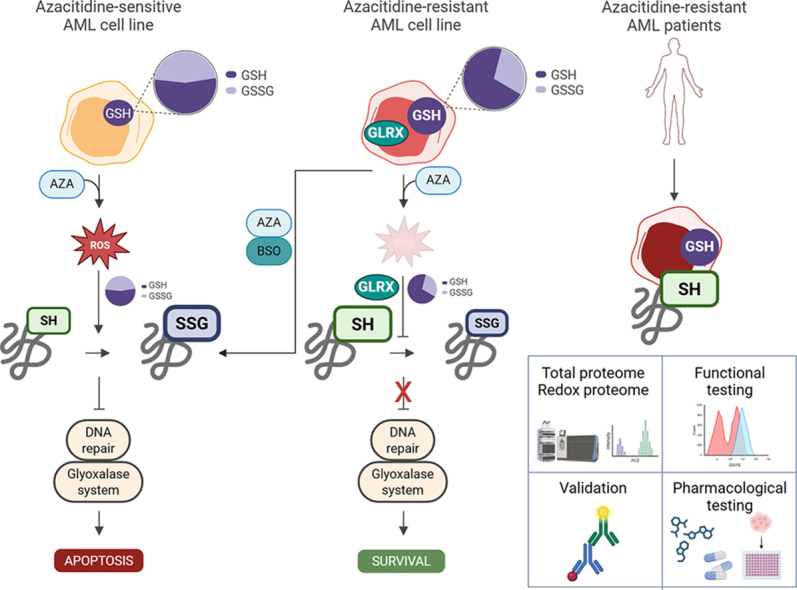

## 1 Introduction

High-risk myelodysplastic syndromes (MDS) often progress to acute myeloid leukemia (AML). The hypomethylating agent azacitidine (5-azacytidine, AZA), nowadays combined with venetoclax (VEN), is the standard first-line treatment for AML patients ineligible for intensive chemotherapy. AZA alone significantly prolongs survival, but the initial response is always followed by the emergence of resistance and disease relapse [1,2]. The combination of AZA with VEN has resulted in favorable responses in treatment-naïve AML patients unfit for intensive chemotherapy, with efficacy superior to azacitidine monotherapy [3,4]. However, the efficacy of VEN is strongly supported by AZA [5], and up to 30 % of AML patients fail to respond to the combined therapy, and almost all treatment responders eventually relapse [6]. Upon therapy failure, AML patients face a poor prognosis with no approved second-line options, which underscores the urgent need to understand the mechanisms of AZA resistance.

AZA is a nucleoside analogue that reactivates silenced tumor suppressor genes via DNA incorporation and inhibition of DNA methyltransferases, predominantly DNMT1 [7]. Additional RNA-dependent and DNA damage–related mechanisms of action have also been described [[8], [9], [10], [11]]. Most mechanisms of AZA resistance described to date mainly involve intrinsic factors of cancer cells, such as AZA transport, its metabolism, and incorporation into nucleic acids [[12], [13], [14], [15]].

Increasing evidence reveals that hypomethylation therapy not only induces the generation of reactive oxygen species (ROS) but also affects their elimination via modulation of glutathione (GSH) levels [[16], [17], [18], [19]]. This positions AZA as a powerful driver of redox metabolic reprogramming, a hallmark already exploited in AZA + VEN and other leukemia therapies [[20], [21], [22], [23]]. Fluctuations in GSH levels affect overall redox balance and, in the proximity to accessible protein thiol groups, mediate reversible oxidative modifications of cysteine (Cys) residues [24,25]. These modifications include the formation of disulfide bonds, S-glutathionylation, sulfenylation, and nitrosylation, among others [24]. Particular attention has been given to protein S-glutathionylation (P-SSG)—the conjugation of GSH to Cys residues—due to its profound impact on protein function, especially within mitochondria [[26], [27], [28]]. Notably, P-SSG of succinate dehydrogenase has been directly linked to leukemia stem cell survival [22].

Thus, identifying the protein targets of Cys oxidative modifications is critical for understanding how redox reprogramming shapes AZA response and resistance. Mass spectrometry (MS)–based redox proteomics offers a powerful tool to study such protein-level signaling, although its technical demands require rapid thiol blocking and efficient removal of unreacted tags. New approaches to labeling and detecting Cys-containing peptides using the iodoacetyl Tandem Mass Tag (iodoTMT) sequential labeling strategy have recently been introduced [29]. This approach was further adapted by us and applied in various cell types, including primary mouse hematopoietic stem and progenitor cells [30].

In this study, we applied iodoTMT-based redox proteomics to map site-specific oxidative modifications of Cys residues and the complementary quantitative proteomics to determine protein abundance in AZA-sensitive and -resistant AML cells. This allowed us to assess the contribution of AZA-induced ROS to AZA toxicity and resistance. The findings were validated by functional assays using pharmacological inhibitors. Our results uncover critical redox-dependent mechanisms of AZA action that can be exploited to overcome chemotherapy resistance.

## 2 Methods

### 2.1 Cell culture

The OCI-M2 cell line (established from a 56-year-old patient with erythroleukemia (AML-M6) representing the end stage of a previously identified myelodysplastic syndrome (DSMZ collection, #ACC 619) was used to generate 5-azacytidine (azacitidine, AZA) resistant cells (R^AZA^). OCI-M2 R^AZA^ subclones were produced by gradually increasing the dose of AZA added to the media (the protocol consisted of the addition of up to 8 μM AZA every 2 days for 6 weeks) as previously described [31]. Cells were cultured at 37 °C, 5 % CO_2_ in a complete Iscove's Modified Dulbecco's Medium containing 20 % (Gibco) fetal bovine serum (FBS) (Biosera) and antibiotics penicillin/streptomycin (Biosera).

### 2.2 Treatments

For the redox proteomic and quantitative proteomic analysis, OCI-M2 AZA-sensitive (S^AZA^) and R^AZA^ cells were incubated with 1 μM AZA (S^AZA^ + AZA, R^AZA^ + AZA) or vehicle (S^AZA^, R^AZA^) for 24 h. The treatment conditions for the detection of ROS and GSH using FACS were as follows: 0.5, 1, 2 μM AZA for 24 h; 500 μM buthionine sulfoximine (BSO), 0.5 μM Erastin (Era), 20 μM 2-AAPA for 2 h. In the WST-1 proliferation assays, 5 μM, 10 μM, and 15 μM Ezatiostat (EZA); a concentration range of AZA (0.01–100 μM) in combination with 100, 500 μM BSO, and 100 μM, 1 mM N-acetylcysteine (NAC); and a concentration range of diamide (0.001–10 mM) were incubated for 72 h. For the real-time proliferation assay, cells were incubated with a concentration range of AZA (0.01–100 μM) and inhibitors of GSH metabolism: 0.5 μM Era, 15 μM BSO, and 20 μM 2-AAPA for 7 days or 50 μM NAC for 8 days. All treatments were performed at 37 °C. For immunodetection, cells were treated with 1 and 2 mM diamide for 15 min; 0.5, 1, 2 μM AZA for 24 h, and 500 μM BSO for 24 h.

### 2.3 WST-1 cell proliferation assay

Half-maximal inhibitory concentration (IC50) of AZA was assessed using a WST-1 cell proliferation assay. 10,000 cells were seeded in a minimum of 3 replicates onto each well of a 96-well plate and treated with a decimal dilution range of an inhibitor/drug or their combination and incubated for 72 h at 5 % CO_2_, 37 °C. After 72 h WST-1 cell proliferation reagent was added, and the samples were incubated for an additional 1 h. The amount of metabolically active cells in the wells was read as absorbance change compared to untreated controls using an ELISA reader (TECAN).

### 2.4 Real-time cell proliferation assay

Real-time cell proliferation was evaluated using Incucyte (Sartorius). 10,000 cells per well were seeded in 2–3 replicates onto a TPP 96-well plate pre-treated with Cell-Tak (Corning) according to the manufacturer's protocol and treated with the desired concentration range of an inhibitor/drug. The cell growth was monitored in a phase channel for 7–8 days with 4 images/well and a scan frequency set to every 4 h. The proliferation activity was expressed as confluence change (mean, SEM) relative to the initial value of each condition.

### 2.5 Primary cells preparation

AML patients used in the study were treated with Vidaza in a regimen of 75 mg/m2, 5 + 2+2 at our institution. Patients' characteristics are specified in Supplementary Table 3. The patient's informed consent and approval of the Institutional ethical committee were obtained under # 1110/20 S-IV, and the observational study was entitled: „Monitoring of the effect of nutritional supplements alongside the therapy with 5-azacytidine in patients with Myelodysplastic syndrome”. Total mononuclear cells were isolated from specimens by standard Ficoll procedures as previously described [32].

### 2.6 Flow cytometry (Intracellular ROS and GSH)

Intracellular ROS and GSH in OCI-M2 S^AZA^ and R^AZA^ cells, bone marrow leukemic cells were detected using the CellROX (Thermo Scientific) and ThiolTracker Violet (Thermo Scientific), respectively, according to the manufacturer's protocols. The samples were analyzed by flow cytometry Fortessa (BD Biosciences, San Jose, CA, USA). All measurements were performed in at least three replicates. Statistical significance was tested by Student's t-test (∗p < 0.05; ∗∗p < 0.01; ∗∗∗p < 0.001, ∗∗∗p < 0.0001) or ANOVA with Dunnett's multiple comparison as specified by each experiment.

### 2.7 Sample preparation for redox proteomic analysis

Sequential redox proteomic analysis was based on differential labelling of free and reversibly oxidized thiols with thiol (SH) reactive isobaric probes iodoTMT (ThermoFirsher Scientific), which allows site-specific identification and quantification of reversibly modified Cys thiols as described [30]. Redox proteomic analyses were performed in three biological replicates for each condition, except for R^AZA^ in two TMT6-plex runs. To enable cross-run comparison, the R^AZA^ condition was included in both runs with three replicates each, resulting in a total of six biological replicates for R^AZA^. For the OCI-M2 cell line, S^AZA^ and R^AZA^ cells were seeded in 3 replicates at 6-well plates at a concentration of 5 x 10^5^ cells per well and cultivated in either complete IMDM medium (control samples) or complete IMDM supplemented with 1 μM 5-azacytidine. After 24 h, the samples were harvested, lysed, and proteins were labelled with iodoTMT as described earlier, with slight modifications [30]. Briefly, naturally occurring free protein thiols were blocked by suspending the pellet in lysis buffer (LB, 3 % sodium deoxycholate (SDC)/200 mM triethylammonium bicarbonate (TEAB)/1 mM ethylenediaminetetraacetic acid (EDTA)) with 4 mM iodoTMT1 and incubated for 2 h at 37 °C. Excess of unreacted iodoTMT1 was removed by precipitation with acetone (1:4), and reversibly oxidized protein thiols were reduced with 5 mM tris(2-carboxyethyl)phosphine (TCEP)/LB for 1 h at 50 °C, followed by blocking of newly occurred free thiols with 4 mM iodoTMT2 for 2 h at 37 °C. Such labelled samples were combined for each replicate into a 6-plex and digested with Trypsin (Promega) (enzyme: protein = 1: 50 w/w) overnight, cleaned up with C18 SePak columns, and analyzed by liquid chromatography with tandem mass spectrometry (LC/MS).

In the redox proteomic analysis of primary bone marrow leukemic cells, three AML patients (4R, 5R, 6R, Supplementary Table 3) were positively enriched for AML blasts with the CD34 MicroBead Kit (Miltenyi Biotech) using the AutoMACS Pro Separator (Miltenyi Biotech). 5 x 10^5^ cells were lysed, and proteins were labelled with iodoTMT and further processed using the same protocol as for the OCI-M2 cell line described above.

### 2.8 Sample preparation for quantitative proteomics

S^AZA^ and R^AZA^ cells were seeded in 3 replicates onto 6-well plates at a concentration of 5 x 10^5^ cells per well and cultivated in either complete IMDM medium (control samples), or complete IMDM supplemented with 1 μM AZA. After 24 h, the samples were harvested and lysed in 0.1 % Rapigest (Waters)/100 mM TEAB. Cys were reduced with 5 mM dithiothreitol (DTT) at 56 °C for 30 min and blocked with a 10 mM iodoacetamide (RT for 30 min in darkness). Samples were digested with Trypsin (Promega) (enzyme: protein = 1:50 w/w) at 37 °C overnight. After digestion, samples were labelled with TMT 16-plex (Thermo Scientific) according to the manufacturer's protocol. The reaction was quenched by 0.2 % hydroxylamine. Samples were multiplexed, Rapigest was precipitated with 1 % TFA, and the sample was cleaned up on C18 SePak columns and analyzed by LC/MS. Samples were fractionated with high-pH reverse-phase fractionation as follows. Labelled samples were resuspended in mobile phase A (5 % of acetonitrile (ACN) mixed with 20 mM NH4FA buffer, pH 10) and loaded onto an analytical C18 column (YMC-Triart C18, 12 nm, 1.9 μM, 0.3 mm ID x 300 mm, YMC America). Peptides were eluted with mobile phase B (80 % of ACN mixed with 20 mM NH4FA buffer, pH 10) gradient from 1 % to 60 % in 64 min. Eluting peptides were monitored by UV detection at 214 nm and separated into 8 fractions from 15 min to 85 min of analysis, changing the position every minute. Collected fractions were evaporated to dryness and further analyzed on LC/MS.

### 2.9 LC/MS analysis of redox proteomic samples

MS analyses of OCI-M2 cells were carried out on an Orbitrap Fusion Tribrid MS instrument (Thermo Scientific) equipped with Dionex liquid chromatography using a 175 min linear gradient separation followed by an MS2 method. Peptides were trapped on an Acclaim PepMap 100C18 column (Acclaim PepMap300, C18, 5 μm, 300 Å Wide Pore, 300 μm x 5 mm, 5 Cartridges), followed by gradient elution of peptides on an EASY-Spray column PepMap RSLC C18 (2 μm particle size, 75 μm inner diameter x 500 mm length, 100 Å pore size) using 0.1 % (v/v) formic acid (FA) in LC-MS grade water (solvent A) and 0.1 % (v/v) FA in ACN (solvent B) as the mobile phases. Peptides were loaded with a constant flow of solvent A at 15 μl/min onto the trapping column and eluted via the analytical column at a constant flow of 300 nl/min. Separated peptides were electro-sprayed into the Orbitrap Fusion Tribrid MS instrument (Thermo Scientific). The full MS scan was performed in the Orbitrap in the range of *m*/*z* 400 to 1600 and at a resolution of 120,000 at full-width-half-max (FWHM) using an automatic gain control (AGC) target of 800,000 and an automatic maximum ion accumulation time. Peptide precursors were isolated by a quadrupole for high-energy collisional dissociation (HCD) at a precursor isolation window width of 0.7 m/z, an AGC of 125,000. Maximum injection time was set to 118 ms and HCD collision energy to 35 %. Fragment ions were detected in orbitrap at 60,000 resolution, first mass was set to *m*/*z* 100.

MS analyses of bone marrow leukemic cells were carried out on an Orbitrap Ascend Tribrid MS instrument (Thermo Scientific) equipped with a Dionex liquid chromatography using a 120 min linear gradient separation followed by an MS2 method. Peptides were trapped on an Acclaim PepMap 100C18 column (Acclaim PepMap300, C18, 5 μm, 300 Å Wide Pore, 300 μm x 5 mm, 5 Cartridges), followed by gradient elution of peptides on an EASY-Spray column PepMap RSLC C18 (2 μm particle size, 75 μm inner diameter x 500 mm length, 100 Å pore size) using solvent A and solvent B as the mobile phases. Peptides were loaded with a constant flow of solvent A at 15 μl/min onto the trapping column and eluted via the analytical column at a flow of 400 nl/min, followed by a flow lowered to 200 nl/min at 6 min. Separated peptides were electro-sprayed into the Orbitrap Ascend Tribrid MS instrument (Thermo Scientific). The full MS scan was performed in the Orbitrap in the range of *m*/*z* 400 to 1600 and at a resolution of 120,000 at FWHM using an AGC target of 400,000 and an automatic maximum ion accumulation time. Peptide precursors were isolated by a quadrupole for HCD at a precursor isolation window width of 0.7 m/z, an AGC of 125,000, and a maximum ion accumulation time of 59 ms, and HCD collision energy set to 38 %. Fragment ions were detected in orbitrap at 30,000 FWHM resolution with enhanced resolution mode for TMT and TMTpro reagents. The first mass was set to *m*/*z* 125.

### 2.10 LC/MS analysis of quantitative proteomic samples

A Nano Reversed phase column (Ion Opticks, Aurora Ultimate TS 25 × 75C18 UHPLC column) was used for LC/MS analysis. Samples were loaded onto the trap column (C18 PepMap100, 5 μm particle size, 300 μm x 5 mm, Thermo Scientific) for 4 min at 18 μl/min loading buffer (LCMS water, 2 % ACN, and 0.1 % trifluoroacetic acid). Peptides were eluted with a gradient 4 %–35 % of solvent B in 149 min. Separated peptides were electrosprayed into the Thermo Orbitrap Ascend Tribrid MS instrument (Thermo Scientific). The full MS scan was performed in the orbitrap in the range of 400 to 1600 m/z and at a resolution of 120,000 (at *m*/*z* 200) at FWHM using an AGC target of 100 %. Peptide precursors were isolated using a quadrupole with a 0.7 m/z isolation width and fragmented by CID at a collision energy of 30 %. MS2 scans were in the Ion Trap with AGC target 130 %, and a maximum injection time set to auto. For the real-time search-based MS3 (RTS-MS3) method, precursor ions were isolated in the Ion Trap. Precursors for MS3 scan were isolated using synchronous precursor selection (SPS) with the number of precursors set to 10, the isolation window set to 0.7 m/z, and the HCD collision energy set to 55 %. The Orbitrap was set to a resolution of 30,000, and a scan range of 100–500 m/z with a maximum injection time of 200 ms. The AGC target for the ion trap was 200 %, with a maximum injection time of 200 ms. For real-time library search, *Homo sapiens* database (downloaded from Uniprot in February 2024, with 20,610 entries) with trypsin digestion was used. Cys carbamidomethylation and TMTpro 16plex modifications on lysine and peptide N-termini were set as static modifications, while methionine oxidation was set as a variable modification. The search was limited to 100 ms with Xcorr of 1.4, dCn of 0.1, precursor ppm of 10, and charge state of 2. Matches to database entries with “##” (reverse) and “contaminant” were excluded. MS3 excluded peaks 25 ppm below the precursor mass width and 25 ppm above, also excluding TMTpro tag loss ions. The total cycle time was 3 s.

### 2.11 MS data analysis and bioinformatic analysis of proteomic data

MS raw data from redox proteomic analyses were processed with Thermo Proteome Discoverer 3.1 (Thermo Scientific) with the following settings: for data from MS2-analyzed reporter ion with quantification of Cys-specific iodoTMT labels reporter ions was used; data were searched using the in-built Sequest search engine against the Swissprot canonical protein database for Homo Sapiens (downloaded 2023.06.28; 25,170 protein entries), together with commonly observed contaminants; an FDR of 1 % was required for identification at protein, site and PSM level; iodoTMT6plex on Cys was set as a fixed modification; methionine oxidation was set as a dynamic modification; trypsin was set as the enzyme in specific digestion mode allowing two missed cleavage sites; precursor and MS/MS fragment mass tolerance was set to 10 ppm and 0.6 Da, respectively. Data was filtered for contaminants and non-Cys peptides. Reporter ion intensities were log2-transformed. Data were normalized by adjusting the median of each channel to the mean of the medians of either free (SH) or oxidized (Sox) thiols, respectively. Normalized log2 data were non-transformed, and the sum of SH and Sox quantitative values were calculated for each ID. Zero values were replaced by “NA” and the oxidation level (%) was calculated as [Sox/(SH + Sox)]∗100. After confirming the normal distribution of the data, the p-value for each peptide was determined using a two-sided Student's t-test. Correction for multiple testing using permutation-based FDR correction (adjusted p < 0.1) implemented in Perseus (version 2.0.11.0) [33] was applied, and if no significant changes were found, significance was evaluated for peptides with p < 0.05. Changes in oxidation level higher than 5 % and with an adjusted p-value < 0.1 or p-value < 0.05 were considered differential. Perseus and GraphPad Prism were used for data visualization. Gene ontology (GO) enrichment analysis was done using the functional annotation tool DAVID [34]. Redundant GO terms were filtered out using REVIGO [35]. GO enrichment of Reactome pathways for primary samples was done in Cluster Profiler in the R package [36]. ReactomeFIViz Cytoscape application was used for the generation of a functionally grouped annotation network [37]. Previously described reversible modifications were annotated from RedoxDB [38]. Known zinc finger domains and GO annotations were retrieved from UniProt (October 10, 2023). The MS proteomics data and Proteome Discoverer output files have been deposited to the ProteomeXchange Consortium via the PRIDE [39] partner repository with the dataset identifier PXD056452.

MS raw data from quantitative proteomic analysis were processed with Thermo Proteome Discoverer 3.1 (Thermo Scientific) with the following settings: abundances were used for protein quantification; data were searched using the in-built Sequest search engine against the Swissprot canonical protein database for Homo Sapiens (downloaded 2023.06.28; 25,170 protein entries), together with commonly observed contaminants; an FDR of 1 % was required for identification at protein, site and PSM level; TMTpro on N-term and lysines, and carbamidomethylation on Cys were set as a fixed modification; methionine oxidation and acetylation of protein N-term were set as a dynamic modification; trypsin was set as the enzyme in specific digestion mode allowing two missed cleavage sites; precursor and MS/MS fragment mass tolerance was set to 10 ppm and 0.6 Da, respectively. Data was filtered for contaminants. Reporter ion intensities were log2-transformed and filtered to contain at least nine valid values. Data were normalized to the median. After confirming the normal distribution of the data, the p-value for each peptide was determined using a two-sided Student's t-test corrected for multiple testing using permutation-based FDR correction implemented in Perseus (version 2.0.11.0) [33]. Changes in log2 abundance with an adjusted p-value < 0.1 were considered differential. Perseus and GraphPad Prism were used for data visualization. The MS proteomics data and Proteome Discoverer output files have been deposited to the ProteomeXchange Consortium via the PRIDE [39] partner repository with the dataset identifier PXD069589.

### 2.12 Western blots

For the detection of phosphorylated Ser139 (p139) of histone H2AX (yH2Ax), cleaved-caspase 3 (c-Casp3), and (cleaved) Poly [ADP-ribose] polymerase 1 ((c)-PARP1, β actin (β-ACT), and α tubulin (α-TUB) cells were collected, 3x washed with PBS and lysed in RIPA lysis buffer (150 mM NaCl, 1 % NP-40, 0.5 % sodium-deoxycholate, 0.1 % sodium dodecyl sulphate, 10 mM Tris-HCl [pH 8]) supplemented with protease and phosphatase inhibitor cocktails (Thermo Scientific) and for detection of P-SSG additionally supplemented with 100 mM N-ethylmaleimide. Lysates were cleared by centrifugation at 15,000×*g* for 10 min at 4 °C and assayed for protein concentration using the BCA Protein Assay Kit (Thermo Scientific). Samples with equal amounts of proteins were incubated for 5 min at 95 °C with loading buffer (BioRad) and with 10 mM DTT (for non-reducing conditions, DTT was replaced with H_2_O) and separated on SDS-PAGE using 10 % MiniProtean TGX Precast Protein Gels (BioRad) and transferred to PVDF membranes (BioRad). Primary antibodies and dilutions: Mouse GSH Monoclonal Antibody (D8) (1:5,000, Thermo Scientific, #MA1-7620), rabbit anti-c-Cas3 antibody (1:2,500, Cell Signalling, #9664), rabbit anti-phospho (Ser139)-H2Ax antibody (1:1000, Cell Signalling, #2577), rabbit anti-PARP1 (1:1000, Cell Signalling, #5942), mouse anti-α-TUB antibody (1:2000, Proteintech, #66031-1-Ig), goat anti-β-ACT antibody (1:5,000, Santa Cruz Biotechnology, #Sc-1616). Secondary antibodies and dilutions: donkey anti-rabbit (1:10,000, Jackson ImmunoResearch, # 711-036-152), goat anti-mouse (1:2000, BioRad, #1706516), rabbit anti-goat (1:10,000, Jackson ImmunoResearch, #305-036-003). Membranes were developed using SuperSignal West Femto Maximum Sensitivity Substrate (Thermo Scientific).

## 3 Results

### 3.1 Redox proteomics identifies redox-sensitive cysteine targets of azacitidine in AML cells

To investigate AZA-triggered redox pathways, we analyzed protein Cys oxidative modifications in AZA-sensitive AML OCI-M2 cells (S^AZA^) (Fig. 1A). We first assessed the redox homeostasis of S^AZA^ cells by detecting levels of ROS and intracellular thiols, mainly GSH, using flow cytometry. AZA treatment significantly elevated both ROS and GSH at 1 μM within 24 h (Fig. 1B and C). Based on these findings, we applied these conditions for MS-based redox proteomics using our established workflow (Fig. 1A) [30].

**Fig. 1:**
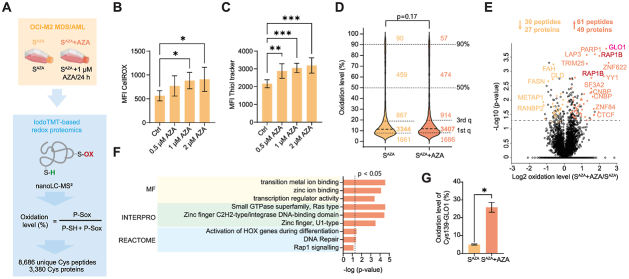
**AZA triggers selective oxidation of redox-sensitive Cys residues in AML cells.** (A) Outline of experimental strategy for site-specific detection of the redox state of protein thiols using sequential Iodoacetyl Tandem Mass Tag (iodoTMT) in S^AZA^ OCI-M2 cells, untreated and treated with 1 μM AZA for 24 h (S^AZA^ + AZA), analyzed by nano liquid chromatography (nanoLC) with tandem mass spectrometry (MS), n = 3 biological replicates. The number of identified and quantified cysteine (Cys) peptides and Cys-containing protein groups is indicated. P-Sox stands for proteins with reversibly modified thiol (SH-) residue, P-SH stands for free or reduced (non-modified) SH- residue. (B–C) Flow cytometry quantification of intracellular reactive oxygen species (B) and glutathione (C) levels in S^AZA^ OCI-M2 cells treated with DMSO (Ctrl) and 0.5 μM, 1 μM, and 2 μM AZA for 24 h. Data are acquired from three biological replicates and two independent measurements. Data represent mean ± SEM. ANOVA with Dunnett's multiple comparison: adjusted p ∗ < 0.05, ∗∗ < 0.01, ∗∗∗ < 0.001. (D) Distribution of average Cys peptide oxidation levels (%) of S^AZA^ and S^AZA^ + AZA, n = 3 biological replicates. The number of peptides within defined intervals is stated. Data represent the mean from three biological replicates. Student's *t*-test of median oxidation level. 1st q - first quartile, 3rd q - third quartile. (E) Volcano plot highlighting significantly (Student's *t*-test p < 0.05 and >5 % change in oxidation) oxidized and reduced Cys-containing peptides between S^AZA^ and S^AZA^ + AZA, n = 3 biological replicates. Glyoxalase system and small GTPases are marked with pink and dark red, respectively. The number of significantly altered Cys peptides and proteins they belong to is indicated. (F) Gene ontology enrichment analysis for overrepresentation of molecular function (MF), protein domains (INTERPRO), and functional pathways (REACTOME) of proteins of AZA-upregulated proteins. The three most significantly changed processes from each category are shown. (G) Oxidation level (%) of Cys139 of protein Lactoylglutathione lyase (GLO1) estimated by redox proteomics. Data represent mean ± SEM, n = 3 biological replicates. Student's *t*-test: ∗p-value < 0.05.

The filtered redox dataset comprised 8,686 unique Cys peptides from 3,380 proteins (Fig. 1A–Supplementary Table 1). Oxidation levels (%) of each peptide were calculated as the fraction of reversibly oxidized thiols (Sox) relative to the sum of reduced (SH) and Sox (Fig. 1A). Replicates showed high reproducibility (Supplementary Fig. 1A), with median coefficients of variation of 7–9 % (Supplementary Fig. 1B). Cys peptide oxidation ranged from 0 % to 99 %, with <100 peptides exceeding 90 % oxidation in both untreated and AZA-treated cells (Fig. 1D). AZA did not trigger proteome-wide oxidation in S^AZA^ cells compared with untreated controls as the median of peptide oxidation level (%) did not differ significantly (Fig. 1D).

We compared peptide oxidation levels across conditions using Student's t-test, selecting events with *p* < 0.05 and > 5 % change in oxidation. We revealed that AZA triggered oxidation of 61 Cys-containing peptides from 49 proteins, while 30 Cys-containing peptides of 27 proteins were significantly reduced in parental OCI-M2 cells (Fig. 1E). GO enrichment analysis of AZA-upregulated proteins highlighted a strong overrepresentation of molecular functions linked to transition metal and zinc ion binding, transcriptional regulation involved in HOX genes activation, DNA repair and signaling (Fig. 1F). The analysis of protein domains revealed that AZA induced oxidation of small GTPases (Supplementary Fig. 1C) or proteins with zinc finger DNA-binding domain (Fig. 1F). Both groups of proteins contain highly conserved redox sensitive Cys residues [40,41]. AZA also markedly affected protein Lactoylglutathione lyase (GLO1), being among the most significantly oxidized proteins (Fig. 1E – magenta label). Specifically, AZA increased oxidation of Cys139 of GLO1 from 5 % to 26 % (Fig. 1G). Overall, these findings indicate that AZA preferentially targets redox-sensitive Cys residues in key regulatory proteins, thereby likely disrupting cellular processes essential for genome stability.

### 3.2 Oxidation of zinc finger proteins impairs DNA repair and triggers apoptosis upon AZA treatment

Detailed analysis of zinc finger proteins exhibiting the most pronounced oxidation shifts upon AZA treatment revealed that majority of them were nucleic acid binding proteins with CCHC and C2H2-type zinc finger domain, which are key regulators of gene expression and DNA repair (Fig. 2A). Network analysis of these proteins revealed functional association with transcription, chromatin organization, and DNA repair (Fig. 2B), suggesting a coordinated, spatially associated oxidation of these proteins by AZA-induced ROS. Among them, AZA increased oxidation of PARP1 at Cys24 by 15 % (Fig. 2C), a modification known to disrupt structural integrity, promote zinc release, impair DNA-binding capacity, and ultimately inactivate DNA repair, thereby triggering cell death [42].

**Fig. 2:**
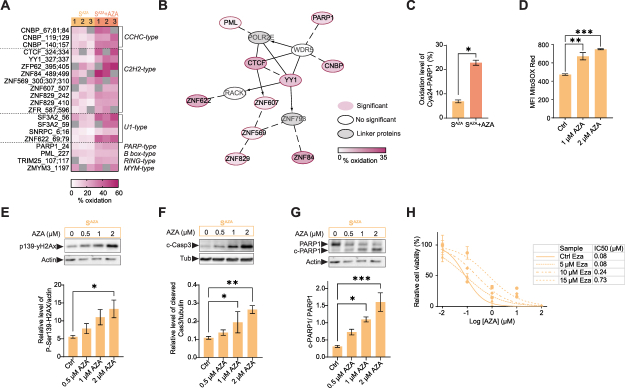
**Oxidative inactivation of zinc finger and glyoxalase proteins links AZA to DNA damage and apoptosis.** (A) Peptides with zinc finger domains exhibited significant oxidation shifts after AZA treatment with 1 μM AZA for 24 h (S^AZA^ + AZA) in S^AZA^ OCI-M2 cells, n = 3 biological replicates (1, 2, 3). The type of zinc finger domains is indicated. Grey fields indicate no quantitative value. (B) Network analysis of zinc finger proteins with AZA-induced oxidation (Student's *t*-test p < 0.05 and > 5 % change in oxidation) showing functional associations in transcription, chromatin organization, and DNA repair. Edges indicate functional interactions, solid line: known functional interaction, dashed line: predicted interaction, arrows: regulation/activation. (C) Oxidation level (%) of Cys24 of Poly [ADP-ribose] polymerase 1 (PARP1) estimated by redox proteomics. Data represent mean ± SEM, n = 3 biological replicates. Student's *t*-test: ∗p-value < 0.05. (D) Flow cytometry quantification of mitochondrial ROS levels in S^AZA^ OCI-M2 cells after 1 μM and 2 μM AZA for 24 h. Data represent mean ± SEM. n = 3 biological replicates. ANOVA with Dunnett's multiple comparison: adjusted p ∗∗ < 0.01, ∗∗∗ < 0.001. (E–G) Immunoblots showing the expression of phosphorylated Ser139 (p139) of histone H2AX (yH2Ax) (E), cleaved-caspase 3 (c-Casp3) (F), and cleaved PARP1 (c-PARP1)/not cleaved PARP1 (G) in S^AZA^ OCI-M2 cells after 0.5 μM, 1 μM and 2 μM AZA for 24 h. Relative quantification of each immunoblot is displayed in a bar graph. Data represent mean ± SEM, n = 3 biological replicates. ANOVA with Dunnett's multiple comparison: ∗p-value < 0.05, ∗∗p-value < 0.01, ∗∗∗p-value < 0.001. (H) Relative proliferation rate of S^AZA^ OCI-M2 cells in the presence of AZA and 5 μM, 10 μM, and 15 μM inhibitor of glutathione-S-transferase (GSTP1) inhibitor Ezatiostat for 72 h. Data represent mean ± SEM, n = 3 biological replicates.

Intriguingly, the oxidation, specifically S-glutathionylation, of Cys139-GLO1 has also been associated with DNA damage-induced cell death. GLO1 is the first enzyme of the glyoxalase system, which catalyzes the conversion of the hemimercaptal formed by GSH and methylglyoxal into (R)-S-lactoylglutathione (sLG), and Hydroxyacylglutathione hydrolase (GLO2), which subsequently hydrolyzes sLG to d-lactate and regenerates GSH [43]. The catalytic activity of GLO1 critically depends on Cys139, where P-SSG inhibits the enzyme, leading to toxic methylglyoxal accumulation, followed by mitochondrial ROS formation, DNA damage, and accelerated apoptosis [44,45].

Consistently, AZA treatment induced mitochondrial ROS production (Fig. 2D) and strong DNA damage signaling, as indicated by Ser139 phosphorylation of histone γH2AX (Fig. 2E). Cleavage of caspase-3 (c-casp3) further confirmed that DNA damage was associated with caspase-dependent apoptosis (Fig. 2F). Activated caspases further cleave PARP1, reinforcing the suppression of DNA repair [46]. In line with this, our data demonstrated that AZA induced PARP1 cleavage (Fig. 2G). Finally, we tested the functional role of P-SSG in the AZA response using the glutathione-S-transferase π (GSTP1) inhibitor Ezathiostat. Inhibition of GSTP1-mediated P-SSG formation increased AZA resistance in S^AZA^ cells in a concentration-dependent manner (Fig. 2H). Collectively, these results show that AZA exposure leads to cell death via oxidation (P-SSG) and deactivation of proteins in S^AZA^ OCI-M2 cells.

### 3.3 AZA-resistant AML cells display impaired redox response and reduced protein S-glutathionylation

Next, we analyzed redox pathways in an AZA-resistant clone of OCI-M2 cells (R^AZA^) with IC50^AZA^ values ranging from 3 to 20 μM, previously developed by us (Minařík et al., 2022). Unlike AZA-sensitive cells, R^AZA^ showed neither ROS production nor GSH induction in response to AZA treatment within the 0.5–2 μM concentration range (Supplementary Fig. 2A and B). The redox proteomic analysis of untreated R^AZA^ and treated with 1 μM AZA for 24 h R^AZA^ cells identified 8,686 unique Cys peptides from 3,380 proteins with high reproducibility (Supplementary Fig. 2C) between replicates and median coefficients of variation of 11–12 % (Supplementary Fig. 2D). Cys peptide oxidation ranged from 0 % to 99 %, with <80 peptides exceeding 90 % oxidation in both untreated and AZA-treated cells, similarly to S^AZA^ (Fig. 3A). AZA did not trigger proteome-wide oxidation in R^AZA^ cells and in contrast to S^AZA^, 48 Cys peptides were reduced by AZA while only 11 Cys peptides were oxidized (Fig. 3B). AZA did not trigger oxidation of previously identified protein groups, such as GTPases, DNA damage-associated proteins, or GLO1 observed in S^AZA^ (Supplementary Fig. 2E). Consistently, AZA induced only mild DNA damage and PARP1 cleavage, without detectable Cas3 activation (Supplementary Fig. 2F).

**Fig. 3:**
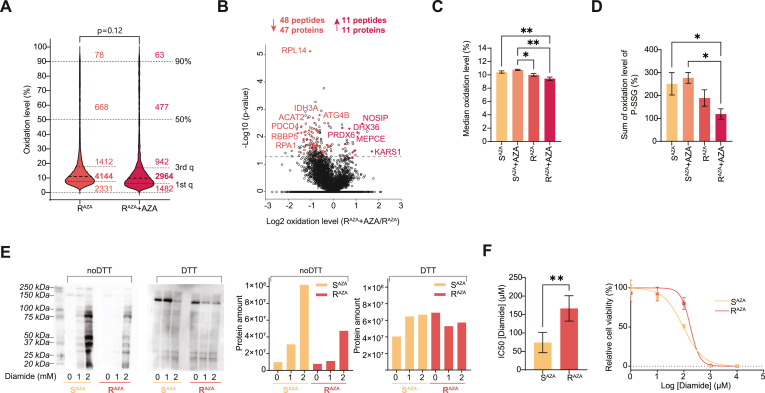
**AZA-resistant AML cells display reduced protein oxidation and impaired S-glutathionylation.** (A) Distribution of Cys peptide oxidation level (%) of R^AZA^ OCI-M2 cells after 1 μM AZA for 24 h (R^AZA^ + AZA). The number of peptides within defined intervals is stated. Student's *t*-test of median oxidation level. Data represent mean, n = 6 (R^AZA^) and 3 (R^AZA^ + AZA) biological replicates. 1st q - first quartile, 3rd q - third quartile. (B) Volcano plot highlighting significantly (Student's *t*-test p < 0.05 and > 5 % change in oxidation) oxidized and reduced Cys-containing peptides upon AZA treatment after 1 μM AZA for 24 h in R^AZA^, n = 6 (R^AZA^) and 3 (R^AZA^ + AZA) biological replicates. The number of significantly altered Cys peptides and proteins they belong to is indicated. (C) Median oxidation levels for all quantified peptides in S^AZA^ and R^AZA^ OCI-M2 cells after 1 μM AZA for 24 h (S^AZA^ + AZA, R^AZA^ + AZA). Data represent mean ± SEM in at least three biological replicates. ANOVA with Fisher's LSD test ∗p < 0.05, ∗∗p < 0.01. (D) Global protein S-glutathionylation (P-SSG) estimated by redox proteomics and mapping to RedoxDB. Data are depicted as mean ± SEM in at least three biological replicates. ANOVA with Fisher's LSD test ∗p < 0.05. (E) Global protein P-SSG estimated by immunodetection in S^AZA^ and R^AZA^ OCI-M2 cells after 1 and 2 μM diamide (Dia) for 15 min upon non-reducing conditions (noDTT) and reducing conditions (DTT). Relative quantification of the immunoblots is displayed in bar graphs. (F) Maximal half inhibitory concentrations (IC50) (left) of diamide in S^AZA^ and R^AZA^ evaluated by WST-1 proliferation assay (right). Data represent mean ± SEM, n = 5 biological replicates.

Intriguingly, the median peptide oxidation level (%) was significantly reduced in AZA-treated R^AZA^ compared to AZA-treated S^AZA^ (10.75 vs. 9.43) (Fig. 3C). We mapped our dataset to the oxidative posttranslational modification database RedoxDB (Sun et al., 2012) and identified 388 Cys-sites, of which 28 were annotated as S-glutathionylated (Supplementary Fig. 2G). Notably, total P-SSG was significantly reduced in AZA-treated R^AZA^ cells compared with parental OCI-M2 (Fig. 3D).

Using immunodetection with an anti-GSH antibody, we validated that global P-SSG was reduced in R^AZA^ compared to S^AZA^ (Fig. 3E). Furthermore, R^AZA^ cells responded to the P-SSG-inducing agent diamide with a less pronounced increase in P-SSG compared to S^AZA^ (Fig. 3E). Intriguingly, R^AZA^ cells displayed lower sensitivity to diamide-induced proliferation inhibition. These data indicate the importance of P-SSG for AZA toxicity (Fig. 3F).

### 3.4 Redox and metabolic reprogramming drive the de-glutathionylation phenotype and AZA resistance in AML cells

We next explored pathways that reprogram R^AZA^ cells toward a de-glutathionylation phenotype. P-SSG refers to the conjugation of GSH to protein thiols to either specifically control critical protein functions or to prevent spontaneous disulfide formation and oxidative damage. While some P-SSG reactions occur spontaneously depending on the ratio of reduced GSH to its oxidized form (GSSG), the majority of both glutathionylation and deglutathionylation processes are strictly regulated by glutathione S-transferases (GST) and glutaredoxins (Grx, Glrx), respectively. Using ThiolTracker, we observed that R^AZA^ cells had significantly higher content of intracellular free thiols (Fig. 4A). A complementary colorimetric assay further showed that R^AZA^ cells maintained more than two-fold higher GSH/GSSG ratio compared to S^AZA^ cells (Fig. 4B). This finding indicates that R^AZA^ cells are prone to spontaneous de-glutathionylation.

**Fig. 4:**
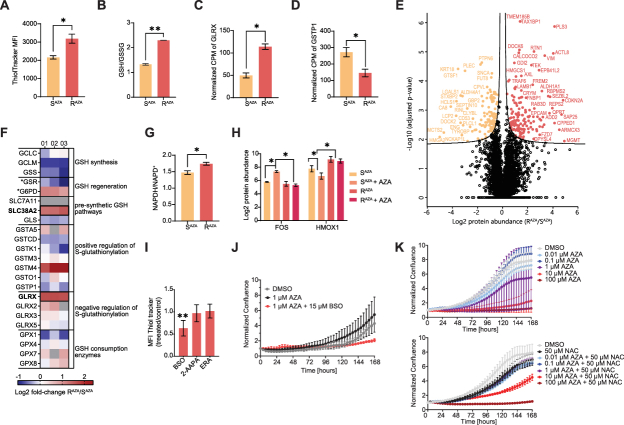
**Enhanced glutathione metabolism and glutaredoxin activity drive the de-glutathionylation phenotype in resistant cells.** (A) Flow cytometry quantification of intracellular GSH levels in AZA-sensitive (S^AZA^) and AZA-resistant (R^AZA^) OCI-M2 cells. Data are acquired from three biological replicates and two independent measurements. Data represent mean ± SEM. Student's *t*-test ∗p-value < 0.05. (B) Colorimetric assay of reduced (GSH) and oxidized (GSSG) glutathione in S^AZA^ and R^AZA^. Data represent mean ± SEM, n = 3 biological replicates. Student's *t*-test, ∗∗p < 0.01. (C, D) Expression level of mRNA for glutaredoxin (GLRX) (C) and Glutathione S-transferase P (GSTP1) (D) in counts per million (CPM) in S^AZA^ and R^AZA^ OCI-M2 cells. Data are depicted as mean ± SD, n = 2 biological replicates. Wald test: ∗adjusted p < 0.05. (E) Volcano plot highlighting proteins with significantly different expression (Student's *t*-test: adjusted p < 0.1) between S^AZA^ and R^AZA^. (F) Heatmap representing expression (log2 (R^AZA^/S^AZA^) of proteins involved in GSH metabolism identified by quantitative proteomics in three replicates (01, 02, 03). Proteins with Student's *t*-test adjusted p < 0.1 are bold and framed, and proteins with Student's *t*-test p < 0.05 are marked with ∗. (G) Ratio of nicotinamide adenine dinucleotide phosphate reduced (NADPH) and oxidized (NADP+) form in S^AZA^ and R^AZA^ estimated by colorimetric assay. Data represent mean ± SEM, n = 3 biological replicates. (H) Relative expression of Protein c-Fos (FOS) and Heme oxygenase 1 (HMOX1) in S^AZA^ and R^AZA^ after 1 μM AZA for 24 h (S^AZA^ + AZA, R^AZA^ + AZA) estimated by quantitative proteomics. Data represent mean ± SEM, n = 3 biological replicates. (I) Flow cytometry quantification of intracellular GSH levels in RAZA OCI-M2 cells treated with GSH metabolism inhibitors (500 μM buthionine sulfoximine (BSO), 20 μM 2-AAPA and 0.5 μM Erastin (ERA)) for 2 h. Data are expressed as a ratio treated to control ± SD, n = 3 biological replicates. Student's *t*-test ∗∗p-value < 0.01. (J) Relative proliferation of R^AZA^ OCI-M2 in the presence of 1 μM AZA and 15 μM BSO for 168 h analyzed by real-time proliferation assay using IncuCyte. Data represent mean ± SEM, n = 2 biological replicates. (K) Relative proliferation of S^AZA^ OCI-M2 in the presence of AZA concentration range (top) and in combination with 50 μM N-acetylcysteine (NAC) (bottom) for 192 h, analyzed by real-time proliferation assay using IncuCyte. Data represent mean, n = 2 biological replicates.

We re-analyzed the transcriptomic data from S^AZA^ and R^AZA^ OCI-M2 clones (Minařík et al., 2022) and found a significant upregulation of glutaredoxin (GLRX) and downregulation of glutathione-S-transferase π (GSTP1) in R^AZA^ (Fig. 4C and D). To investigate the protein levels of glutaredoxins and GSTs, we performed quantitative proteomic analysis of control and AZA-treated S^AZA^ and R^AZA^ subclones of AML cells. The total proteome dataset comprised 7,759 unique protein groups identified and quantified in at least three replicates (Supplementary Table 2). AZA induced changes in the expression of 171 proteins (Student's t-test, p < 0.05) in S^AZA^, whereas in R^AZA^, only 20 proteins were altered (Supplementary Table 2). These data validate our AZA-resistance model. Interestingly, the data further revealed that R^AZA^ had a significantly distinct proteome signature from S^AZA^, displaying differential expression of 278 proteins (Student's t-test adjusted p < 0.1) (Fig. 4E). Examination of proteins involved in the metabolism of AZA confirmed that resistance to AZA was not caused by changes in the expression of these proteins between S^AZA^ and R^AZA^ (Supplementary Fig. 3A). In contrast analysis of proteins associated with GSH metabolism and P-SSG revealed a significant upregulation of GLRX in R^AZA^ cells (Fig. 4F). Further inspection identified Sodium-coupled neutral amino acid symporter 2 (SLC38A2) to be upregulated in R^AZA^ (Student's t-test adjusted p < 0.1). Increased expression of SLC38A2 has been associated with enhanced glutamine uptake, thereby supplying substrates for GSH biosynthesis. At the same time, Glucose-6-phosphate 1-dehydrogenase (G6PD) displayed higher expression in R^AZA^ compared to S^AZA^ using less stringent testing (Student's t-test p < 0.05) (Fig. 4F), which was in line with the elevated ratio of reduced to oxidized nicotinamide adenine dinucleotide phosphate (NADPH/NADP^+^) in R^AZA^ relative to S^AZA^ (Fig. 4G). Together, these findings suggest that the proteomic program of R^AZA^ cells supports biosynthesis of reducing agents such as GSH and NADPH, thereby contributing to the de-glutathionylation phenotype of R^AZA^ cells.

The proteomic analysis further revealed that the redox-responsive transcription factor c-Fos (FOS) was among the most strongly AZA-induced proteins in S^AZA^, yet its level was more than fourfold lower in AZA-treated R^AZA^ compared to AZA-treated S^AZA^ (Fig. 4H). In contrast, the stress-response protein Heme oxygenase 1 (HMOX1), which was strongly downregulated in S^AZA^ upon AZA exposure, remained strikingly elevated in R^AZA^ cells (Fig. 4H). These findings indicate that AZA-sensitive and -resistant cells employ fundamentally distinct redox programs, with FOS and HMOX1 emerging as key adaptive markers. These data further underpin the proteomic signature of R^AZA^ cells, suggesting that their distinct oxidative stress response contributes to AZA resistance compared to S^AZA^.

To assess the role of GSH in AZA response, we inhibited distinct steps of GSH biosynthesis and recycling in R^AZA^ cells (Supplementary Fig. 3B). Buthionine sulfoximine (BSO), an inhibitor of glutamate–cysteine ligase, markedly depleted GSH levels (Fig. 4I) and strongly sensitized R^AZA^ cells to AZA (Fig. 4J, Supplementary Fig. 3D). In contrast, inhibition of GSH reductase with 2-AAPA or blockade of cystine uptake with Erastin failed to alter AZA sensitivity in R^AZA^ cells (Supplementary Fig. 3C). Notably, supplementation of GSH precursor N-acetylcysteine (NAC) markedly decreased sensitivity to AZA of AZA-responsive cells (Fig. 4K, Supplementary Fig. 3E). Interestingly, depletion of GSH with BSO together with AZA enhanced P-SSG in R^AZA^ (Supplementary Fig. 3F), further confirming the link between GSH levels and P-SSG in resistant cells. Collectively, these results suggest that the inefficient P-SSG contributes to AZA resistance.

Our data demonstrates that differential GSH metabolism and P-SSG underpin AZA sensitivity.

### 3.5 Patient-derived AML cells resistant to AZA reflect the redox features of the resistant phenotype

**Fig. 5:**
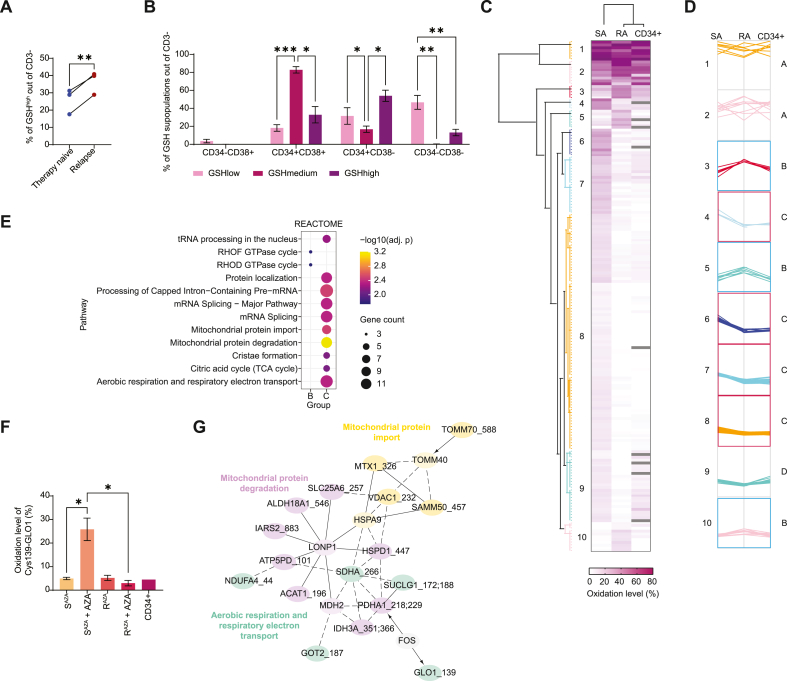
**Patient-derived AML samples validate the mitochondrial and redox signature of AZA resistance.** (A) Flow cytometry analysis of glutathione (GSH) high subpopulations in bone marrow live CD3^−^cells from therapy naïve AML patients and paired samples following Vidaza treatment and relapse, n = 3 biological replicates. Paired Student's *t*-test ∗∗p < 0.01. (B) Distribution of leukemic cells subpopulations in therapy naïve AML patients and paired samples within GSHlow, GSHmedium, and GSHhigh fractions. Data represent mean ± SEM, n = 4 biological replicates. Student's *t*-test ∗p < 0.05, ∗∗p < 0.01, ∗∗∗p < 0.001. (C) Clustering analysis of oxidation level (%) of Cys peptides in leukemic cells of AML patients (CD34^+^) and significantly (adjusted p < 0.1 and >5 % change in oxidation) distinct Cys peptides between AZA-treated AZA-resistant (RA) and AZA-treated AZA-sensitive (SA) OCI-M2 cells. Data represent mean, n = 3 biological replicates. (D) Plot displays the separation of Cys peptides into 10 clusters based on their oxidation profile in SA, RA, and CD34^+^. Clusters with similar oxidation profiles are marked with letters A-D. Red frames indicate protein profiles with reduced cysteines in CD34^+^ cells and the R^AZA^ cell line compared to S^AZA^. Blue frames indicate protein profiles with reduced cysteines in CD34^+^ cells and the S^AZA^ cell line compared to R^AZA^. Data represent mean, n = 3 biological replicates. (E) Gene ontology enrichment analysis of Cys proteins from all groups (A–D) for Reactome pathways. (F) Oxidation level (%) of Cys139 of protein Lactoylglutathione lyase (GLO1) estimated by redox proteomics in S^AZA^ and R^AZA^ after 1 μM AZA for 24 h (S^AZA^ + AZA, R^AZA^ + AZA) and CD34^+^ bone marrow cells of AML patients (CD34^+^). Data represent mean ± SEM, n = at least 3 biological replicates. Student's *t*-test: ∗p-value <0.05. (G) Functional network of proteins from group C enriched for Mitochondrial protein degradation, Mitochondrial protein import, Aerobic respiration, and Electron transport. Edges indicate functional interactions, solid line: known functional interaction, dashed line: predicted interaction, arrows: regulation/activation. Nodes without indicated Cys are linker proteins.

To transfer our findings from the AML cell line *in vivo*, we collected bone marrow samples from high-risk therapy naïve AML patients (1 N, 2 N, 3 N) and paired samples who underwent treatment with Vidaza (s.c. 75 mg/m2, 7 days/cycle) and relapsed (1R, 2R, 3R) (Supplementary Table 3). Flow cytometry analysis showed that cells segregated into distinct subpopulations according to GSH content (GSH^low^, GSH^medium^, GSH^high^; L, M, H) (Supplementary Fig. 4A). While GSH^low^ and GSH^medium^ population did not significantly change with progression of the disease, GSH^high^ population markedly increased upon relapse (Fig. 5A, Supplementary Fig. 4B). Further characterization using CD34 and CD38 antibodies demonstrated that GSH^medium^ fraction was significantly enriched in more differentiated cells (CD34^+^CD38^+^), whereas GSH^high^ fraction was predominantly composed of leukemic stem cells (CD34^+^CD38^−^) (Fig. 5B).

To assess whether the redox state of proteins in AML patients resistant to AZA reflects our observations in AML cell lines, we conducted redox proteomic profiling of CD34^+^ cells isolated from three patients at relapse following prior Vidaza treatment (4R, 5R, 6R) (Supplementary Table 3). Most of the CD34^+^ fraction was represented by the GSH^high^ subpopulation (Supplementary Fig. 4C), thus corresponding to the profile of the AZA-resistant OCI-M2 cell line. In total, we quantified the redox state of 8,722 peptides, filtered to include those with at least one of three quantitative values (Supplementary Table 4). The median proteome oxidation level in CD34^+^ AML cells averaged 7 %, with a range of 0.2 %–98 % (Supplementary Fig. 4D). This represents a lower oxidation level compared with the 10 % observed in the OCI-M2 cell line and is consistent with the reduced oxidation (9 %) measured in AZA-resistant AML cells after AZA treatment (R^AZA^ + AZA) (Fig. 3C). Notably, the peptide redox state of primary patient cells correlated with both AZA-treated S^AZA^ (R = 0.67) and AZA-treated R^AZA^ (R = 0.71) (Supplementary Fig. 4E).

To identify proteins with a redox state like that of resistant cells and to distinguish those that do not react with AZA through oxidation, we mapped the redox proteome of primary cells onto the differentially oxidized proteins between AZA-treated R^AZA^ and S^AZA^ in the OCI-M2 cell line. We identified 293 Cys belonging to 207 proteins with significantly different redox state of their Cys between S^AZA^ and R^AZA^ after AZA treatment using Student's t-test, selecting events with adjusted *p* < 0.1 and > 5 % change in oxidation (Supplementary Fig. 4F). In CD34^+^ AML cells, 218 Cys of 156 proteins overlapped with 207 proteins exhibiting differential Cys redox states between AZA-treated S^AZA^ and R^AZA^ (Fig. 5C). Hierarchical clustering analysis revealed that proteins from bone marrow samples clustered together with R^AZA^, indicating a high overlap of proteins with similar redox states (Fig. 5C). The analysis distinguished ten clusters based on the protein oxidative state (Fig. 5D).

We grouped proteins from clusters with similar profiles into four categories (Fig. 5D, groups A–D). Group B (clusters 3, 5, and 10) included proteins whose cysteines in CD34^+^ cells displayed an oxidative state comparable to S^AZA^ (Fig. 5D, blue frame). In contrast, group C (clusters 4, 6, 7, and 8) comprised proteins with reduced cysteines in CD34^+^ cells, resembling the resistant R^AZA^ cell line (Fig. 5D, red frame). GO enrichment of all groups of proteins (A-C) revealed that pathways enriched in group C were unique and did not overlap with those from other groups (Fig. 5E). These proteins were mainly involved in mitochondrial metabolism, mitochondrial protein import and degradation, as well as mRNA processing and splicing, with mitochondrial pathways being the most significantly enriched (Fig. 5E). GLO1 was identified in CD34^+^ cells in its active form (with reduced Cys 139), like R^AZA^ (Fig. 5F). Interestingly GLO1 was functionally associated with mitochondrial proteins in group C via the transcription factor FOS, which was found to be significantly downregulated in AZA-resistant OCI-M2 cells (Fig. 5G). These findings highlight the role of the glyoxalase system in AZA resistance and suggest a redox signaling link between mitochondrial protein degradation and the glyoxalase system.

## 4 Discussion

This study provides the first comprehensive redox-proteomic characterization of AZA response in AML. Complex identification of oxidative posttranslational modifications of proteins in AML cell lines and in AML patients revealed that AZA selectively targets redox-sensitive Cys residues in regulatory proteins through P-SSG, leading to disruption of DNA repair, accumulation of toxic metabolites, and apoptosis in sensitive AML cells. Resistance to AZA was mediated by a shift towards a reductive redox program, with enhanced GSH metabolism, elevated GLRX activity, and reduced global P-SSG, and can be reverted by modulation of GSH homeostasis. The findings were validated in AML patient samples, underscoring the clinical relevance of redox rewiring.

We found that AZA significantly affected the redox environment of AML cells, characterized by elevated ROS (Fig. 1B). This is consistent with previous reports documenting that anti-leukemic therapies induce oxidative stress [19,[47], [48], [49]]. Here, we provide the first evidence that AZA promoted protein oxidative modifications, selectively oxidizing Cys residues in DNA/RNA binding proteins with zinc finger domain (Fig. 2A) and metabolic enzymes (GLO1) (Fig. 1G). Since oxidation of zinc binding Cys residues can release zinc from these domains and impair DNA-binding activity [41,42,50], our results support the notion that such modifications compromise DNA damage repair in AML cells (Fig. 2E). One of the strongest candidates for this redox regulation was PARP1, where we observed profound oxidation of Cys24 upon AZA treatment (Fig. 2C). Previous studies linked Cys24 modification to the loss of DNA repair capacity [42]. Accordingly, AZA-sensitive cells accumulated severe DNA damage, while resistant cells remained unaffected, with Cys24 oxidized to 7 %. We therefore hypothesize that Cys24 oxidation may function as a molecular switch converting the DNA repair mechanism into an apoptotic trigger. Moreover, AZA-driven oxidation appears to occur in close proximity to DNA incorporation sites, as additional proteins (YY1, CTCF, and PML) (Fig. 2B), known to act in concert with PARP1 to maintain genome stability [[51], [52], [53]], were also oxidized by AZA. Collectively, these findings expand our understanding of AZA's mechanism of action, revealing a previously unrecognized role in redox-dependent regulation of DNA repair, ultimately destabilizing the genome and promoting apoptosis.

In our data, Cys139 of GLO1 demonstrated the strongest AZA-induced oxidation in sensitive versus resistant cells (Fig. 5F). Oxidation of GLO1 at Cys139 was previously recognized as a P-SSG that deactivates the enzyme [44] and has been linked to DNA damage and apoptosis in cancer cells [54,55]. Consistent with this study, pharmacological inhibition of GSTP1-mediated P-SSG using Ezathiostat significantly increased the survival of parental cells in the presence of AZA (Fig. 2H). Taken together, our results point to the glyoxalase system as a potential mediator of AZA response and resistance, warranting further investigation.

Similarly to zinc finger proteins, AZA failed to induce oxidation of GLO1 in AZA-resistant cells. Instead, resistant cells displayed a reductive shift in redox metabolism, characterized by an elevated GSH/GSSG ratio and increased GLRX activity. This observation is consistent with evidence that resistance to anti-cancer therapies can be accompanied by a “redox reset,” in which cells adapt to oxidative stress by reprogramming redox balance [56]. Our findings suggest that resistant cells either effectively prevent the formation of oxidative modifications or efficiently remove them once they occur [27,57,58]. Supporting this, proteomic mapping to RedoxDB [38] revealed that AZA-treated sensitive cells accumulated more S-glutathionylated proteins compared with resistant cells, while global P-SSG was markedly suppressed in resistant cells (Fig. 3D, Supplementary Fig. 2E). Importantly, this phenotype does not appear to be genetically encoded, as our previous genomic profiling of resistant OCI-M2 cells did not uncover mutations directly linked to stress response pathways [31].

Pharmacological inhibition of GSH metabolism further highlighted the importance of redox balance in AZA response. Among the tested compounds, only blockade of de novo GSH synthesis effectively depleted intracellular GSH and resensitized resistant cells to AZA (Fig. 4I and J). This result is consistent with prior studies showing that GSH depletion promotes P-SSG and apoptosis by shifting the GSH/GSSG ratio toward a more oxidative state [59].

Several studies have suggested that therapy resistance in AML and MDS is associated with enhanced antioxidant defenses and metabolic adaptation [60,61]. Our analysis of primary AML samples at relapses following AZA treatment extends these observations by demonstrating a reductive phenotype that mirrors the redox profile of resistant AML cell lines. In line with prior reports linking GSH metabolism to leukemic stem cell survival [22], we observed that resistant patient samples exhibited reduced P-SSG and maintained GLO1 in its active form, thereby preserving redox homeostasis and preventing AZA-induced protein inactivation. Together with our functional data showing that GSH depletion resensitizes resistant cells to AZA, these findings highlight GSH metabolism as a clinically relevant driver of resistance, consistent with and extending earlier evidence that redox adaptation underpins therapy failure in AML. We recognize that correlation analysis alone cannot establish causality. Further functional experiments are therefore needed to confirm whether protein modifications directly contribute to drug resistance, for example, by gene editing of critical Cys and testing the resulting changes in cellular sensitivity to AZA.

Our results point to a critical link between redox homeostasis, cellular metabolism, and mitochondrial integrity (Fig. 5G). Mitochondrial architecture, particularly proteins of the outer mitochondrial membrane, is essential for the proper function and localization of the BCL-2 family members that regulate mitochondrial apoptosis and determine cellular sensitivity to BH3-mimetic VEN [62]. Disruption of mitochondrial structure or metabolic balance may therefore shift the apoptotic threshold by altering the interaction between pro- and anti-apoptotic BCL-2 proteins [62]. The identification of AZA resistance associated with mitochondrial structure and metabolic pathways supports the rationale for combining AZA with BCL-2 inhibition, deepens our understanding of AZA's contribution to the combined therapy, and reveals potential new mitochondrial targets to enhance the therapeutic efficacy of AZA + VEN in AML.

## 5 Conclusions

In conclusion, this study provides the first comprehensive redox-proteomic characterization of AZA response in AML. We identify P-SSG as a previously unrecognized mechanism underlying AZA-induced cytotoxicity, selectively targeting redox-sensitive Cys residues in DNA repair and metabolic enzymes. Importantly, we demonstrate that resistance arises through metabolic reprogramming of GSH homeostasis, favoring de-glutathionylation and protecting critical proteins from inactivation. Importantly, functional modulation of GSH levels directly alters AZA sensitivity, establishing GSH metabolism as a targetable pathway to overcome resistance. Finally, validation in primary AML patient samples underscores the translational relevance of our findings and highlights redox rewiring as a clinically meaningful determinant of relapse. We therefore anticipate that this work will not only provide a deeper understanding of how redox pathways reshape leukemia cells during chemotherapy but will also result in a novel strategy to prevent the emergence of drug resistance.

## Study limitations

The main drawback of our study is the demonstration of the AZA-resistant phenotype using only a single AML cell line. Although we developed AZA-resistant MOLM13 and SKM1 cell lines, showing an ∼8-fold and ∼2-fold increase in IC50, respectively, we did not observe significant differences in redox state between the resistant and parental clones. Notably, these cell lines also differed in their basal GSH content compared with OCI-M2. While MOLM13 cells responded to AZA with up to a 4-fold depletion of GSH, SKM1 GSH levels remained unchanged. In contrast to MOLM13 and SKM1, OCI-M2 carries a TP53 mutation. Patients with TP53 mutations often show strong initial responses to AZA, but resistance develops rapidly, and overall prognosis remains poor. This phenotype is thought to reflect the disrupted stress response and impaired apoptotic regulation characteristic of TP53-mutated cells.

## Declaration of generative AI and AI-assisted technologies in the manuscript preparation process

During the preparation of this work, the authors used Grammarly and ChatGPT to edit the text of the manuscript. After using these tools, the authors reviewed and edited the content as needed and took full responsibility for the content of the publication.

## CRediT authorship contribution statement

**Dušan Nemes:** Data curation, Formal analysis, Methodology, Validation, Writing – review & editing. **Michaela Myšáková:** Formal analysis, Validation, Writing – review & editing. **Lubomír Minařík:** Data curation. **Anna Jonášová:** Data curation. **Tomáš Stopka:** Conceptualization, Project administration, Resources, Supervision, Writing – review & editing. **Kristýna Gloc Pimková:** Conceptualization, Data curation, Funding acquisition, Investigation, Supervision, Visualization, Writing – original draft.

## Declaration of competing interest

The authors declare that they have no known competing financial interests or personal relationships that could have appeared to influence the work reported in this paper.

## Figures and Tables

**figs1:**
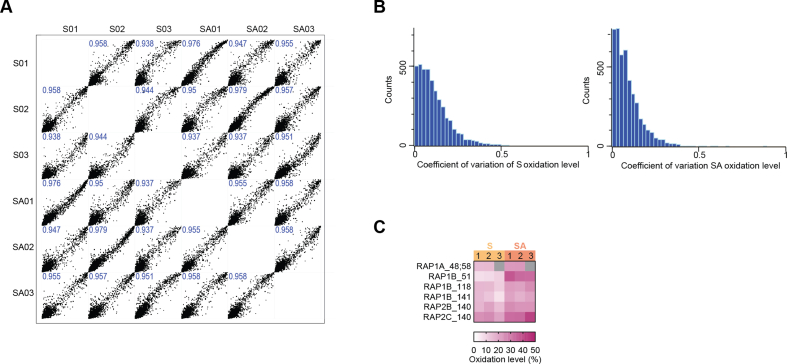


**figs2:**
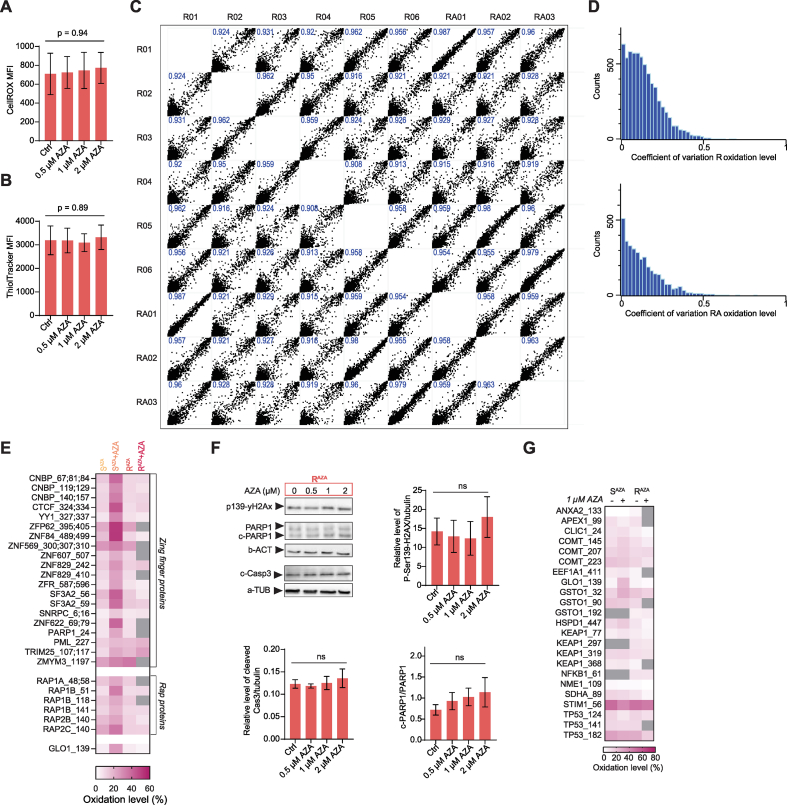


**figs3:**
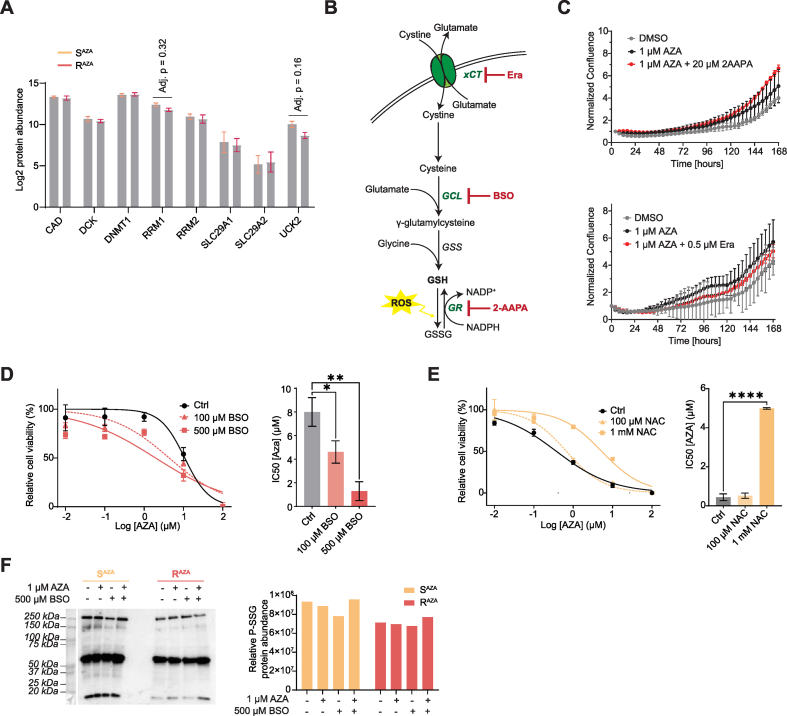


**figs4:**
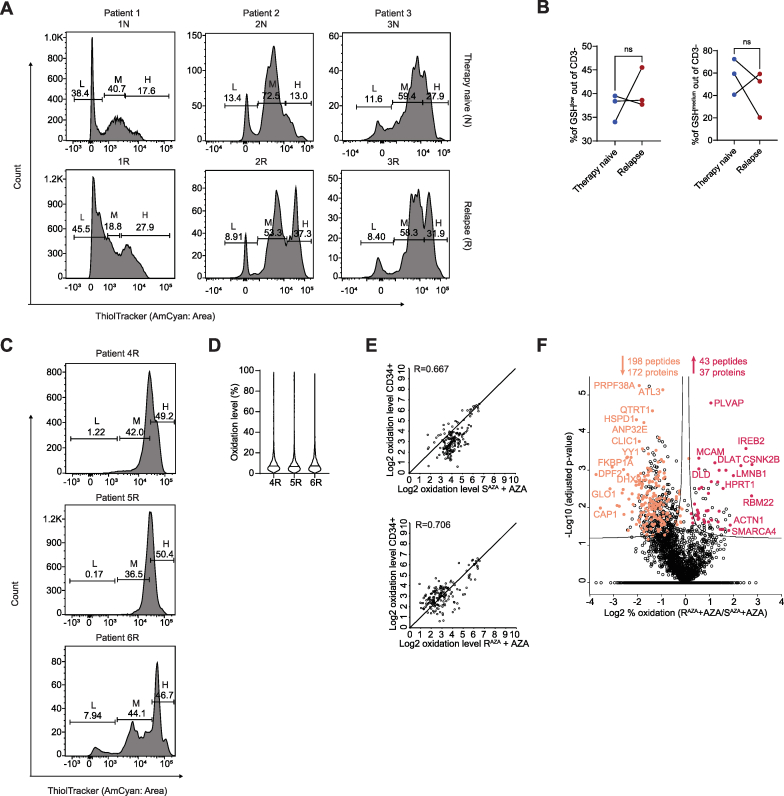


## Data Availability

All proteomic data have been uploaded to ProteomeX and will be fully accessible. References and accession details for each dataset are provided in the Methods section.
